# mGlu2 mechanism-based interventions to treat alcohol relapse

**DOI:** 10.3389/fphar.2022.985954

**Published:** 2022-09-15

**Authors:** Valentina Vengeliene, Rainer Spanagel

**Affiliations:** ^1^ Institute of Psychopharmacology, Central Institute of Mental Health, Faculty of Medicine Mannheim, University of Heidelberg, Heidelberg, Germany; ^2^ Department of Neurobiology and Biophysics, Institute of Biosciences, Life Sciences Center, Vilnius University, Vilnius, Lithuania

**Keywords:** alcohol addiction, craving, relapse, tolerance, DSM-based rat model, metabotropic glutamate receptors, positive allosteric modulators

## Abstract

Recently we identified a deficiency in metabotropic glutamate receptor 2 (mGlu2) function in the corticoaccumbal pathway, as a common pathological mechanism underlying alcohol-seeking and relapse behavior. Based on this mechanism, we hypothesized that mGlu2/3 agonists and mGlu2 positive allosteric modulators (PAMs) may be effective in reducing relapse-like behavior. Two mGlu2/3 agonists, LY379268 and LY354740 (a structural analog of LY379268 six-fold more potent in activating mGlu2 over mGluR3), were tested in a well-established rat model of relapse, the alcohol deprivation effect (ADE) with repeated deprivation phases. Since these agonists do not readily discriminate between contributions of mGlu2 and mGluR3, we also tested LY487379, a highly specific PAM that potentiates the effect of glutamate on the mGlu2 with less specificity on other mGlu receptor subtypes. Both LY379268 and LY354740 significantly and dose-dependently reduced the expression of the ADE. No significant changes in water intake, body weight and locomotor activity were observed. Importantly, repeated administration of mGlu2/3 agonist did not lead to tolerance development. mGlu2 PAM LY487379 treatment significantly reduced expression of the ADE in both male and female rats. Combination treatment of mGlu2/3 agonist and PAM had similar effect on relapse-like drinking to that seen in mGlu2/3 agonist treatment alone. Together with other preclinical data showing that PAMs can reduce alcohol-seeking behavior we conclude that mGlu2 PAMs should be considered for clinical trials in alcohol-dependent patients.

## Introduction

Preclinical studies revealed contribution of metabotropic glutamate receptor 2 (mGlu2) to a variety of neuropsychiatric diseases including alcoholism. Alcohol dependent rats and humans show a pronounced down-regulation of mGuR2, especially in prefrontal regions (Meinhardt et al., 2013; Meinhardt et al., 2021). Very recently, a deficiency in mGlu2 function in the corticoaccumbal pathway was identified as a pathological mechanism that is necessary and sufficient for increased alcohol-seeking behavior (Meinhardt et al., 2021). Alcohol-seeking responses can be best assessed in the reinstatement paradigm. In a typical reinstatement experiment, an animal is trained to self-administer alcohol and the behavior is then subjected to extinction. When the animal reaches some criterion of decreased responding, a conditioned stimulus is said to reinstate alcohol-seeking behavior when it results in renewed responding in the absence of any further response-contingent alcohol reinforcement (Bossert et al., 2013; Spanagel, 2017).

If mGuR2 deficiency is a pathological key mechanism in alcoholism (Holmes et al., 2013; Meinhardt et al., 2013; Meinhardt et al., 2021; Spanagel et al., 2013; Spanagel and Vengeliene, 2013) pharmacological logic dictates that mGlu2 activation should reduce addiction-related behaviors such as alcohol-seeking responses (i.e., craving). Indeed, suppression of cue-induced reinstatement of alcohol seeking by the mGlu2/3 receptor agonist LY379268 was demonstrated by Bäckström and Hyytiä (2005). This initial finding was replicated several times (Zhao et al., 2006; Kufahl et al., 2011; Meinhardt et al., 2021) and extended by showing that stress-induced reinstatement was also suppressed by LY379268 (Zhao et al., 2006; Sidhpura et al., 2010). LY379268 was even more effective in reducing cue- and stress induced reinstatement of alcohol-seeking behavior in rats with a history of alcohol dependence (post-dependent state, Meinhardt and Sommer, 2015) than in non-dependent rats (Sidhpura et al., 2010; Kufahl et al., 2011). Rodd et al. (2006) also reported a reduction of alcohol-seeking response following pharmacological blockade of mGlu2/3 in alcohol-preferring P female rats. This is a surprising finding because P rats are homozygous for a Grm2 stop codon that leads to largely uncompensated loss of mGlu2 (Zhou et al., 2013).

However, orthosteric group II mGluR (mGlu2/3) agonists, such as LY379268, do not well discriminate between mGlu2 and mGluR3 subtypes and may also be associated with multiple unwanted effects characteristic to directly acting agonists (Marino and Conn, 2006). To overcome these limitations, positive allosteric modulators (PAMs) selective for mGlu2 were recently developed (Marino and Conn, 2006; Caprioli et al., 2018). These PAMs exert their effects through allosteric binding sites of the mGlu2s receptor and selectively activate the receptor in the presence of glutamate (Schaffhauser et al., 2003). Hence, it was shown that glutamate only partially stabilizes the extracellular domains of mGlu2 in the active state, whereas full activation is only observed in the presence of a PAM (Cao et al., 2021). AZD8529, a highly specific PAM that potentiates the effect of glutamate on the mGlu2, was recently shown to effectively reduce cue-induced alcohol-seeking responses in the reinstatement paradigm (Augier et al., 2016).

Taken together, these results identify mGlu2 as a target for medication development for alcoholism. Given that during withdrawal and protracted abstinence, alcohol-seeking responses can result in relapse, we hypothesize that mGlu2 agonists can be applied for pharmacological interventions targeting the described mGlu2 deficit and reducing relapse. For this purpose, we used an established rat model of relapse-the alcohol deprivation effect (ADE) model-to test if the mGlu2/3 agonist LY379268 and LY354740, a structural analog of LY379268 with a six times higher ability to discriminate between mGlu2 and mGluR3, would affect relapse-like drinking. Alcohol relapse can be best measured in the alcohol deprivation effect (ADE) model in long-term alcohol drinking Wistar rats. In this model, renewed access to alcohol solutions after some days of deprivation can lead to a pronounced increase in voluntary alcohol intake in rats. The ADE lasts only for a few days and resembles a relapse situation in alcohol dependent patients. Following repeated deprivation phases, the ADE is characterized by an increased demand for alcohol that results in compulsive drinking behavior (Spanagel and Hölter, 1999; Vengeliene et al., 2009; Vengeliene et al., 2014; Vengeliene et al., 2016; Spanagel, 2017; Foo et al., 2022).

Anti-relapse treatment with a mGlu2/3 agonist would most likely require long-term treatment but it has been reported that chronic administration of group II mGlu receptor agonists can induce robust tolerance (Jones et al., 2005; Bespalov et al., 2016). Therefore, subsequent ADE measurements were performed to test for the development of tolerance and for studying persistent treatment effects in a drug-free period. In the final experiment, we also tested LY487379, a highly specific PAM that potentiates the effect of glutamate on the mGlu2 with little effect on other mGlu receptor subtypes (Schoepp et al., 1997). We tested PAM in the ADE model and also evaluated the potential for sexually dimorphic effects, since sex differences are often seen in the efficacy of alcoholism treatment (Sanchis-Segura and Becker, 2016; Spanagel, 2017).

## Materials and methods

### Animals

Eighty-six two-months-old male Wistar rats and 18 female rats (from our own breeding colony at the CIMH, Mannheim, Germany) were used for the ADE experiments. All animals were housed individually in standard rat cages (Ehret, Emmendingen, Germany) under a 12 h artificial light-dark cycle (lights on at 7:00 a.m.). Standard laboratory rat food (Ssniff, Soest, Germany) and tap water were provided *ad libitum* throughout the experimental period (unless stated otherwise). Body weights were measured weekly. All experimental procedures are approved by the Committee on Animal Care and Use (Regierungspräsidium Karlsruhe, Germany) and were carried out in accordance with the local Animal Welfare Act and the European Communities Council Directive of 24 November 1986 (86/609/EEC).

### Drugs

Alcohol drinking solutions were prepared from 96% ethanol (Sigma-Aldrich, Taufkirchen, Germany) and then diluted with tap water. LY354740 (+) -2-aminobicyclo [3.1.0] hexane-2,6dicarboxylate) (generously provided by Merz Pharmaceuticals, Germany) and LY379268 (-)-2-oxa-4-aminobicyclo [3.1.0]hexane-4,6-dicarboxylate (Tocris Bioscience, United Kingdom) were dissolved in water for injections (aqua ad iniectabilia, Braun, Melsungen AG, Germany). LY487379 N-(4-(2-Methoxyphenoxy)-phenyl-N-(2,2,2-trifluoroethylsulfonyl)-pyrid-3-ylmethylamine (generously provided by AbbVie Deutschland GmbH and Co. KG, Germany) was suspended in water for injections containing 0.5% methylcellulose (Sigma-Aldrich, Germany). All drugs were freshly prepared and injected as a volume of 3 ml/kg intraperitoneally (IP). Control animals received an equal volume of respective vehicle.

### Long-term voluntary alcohol consumption with repeated deprivation phases

As described in Vengeliene et al. (2018) after 2 weeks of habituation to the animal room, rats were given *ad libitum* access to tap water and to 5, 10 and 20% ethanol solutions (v/v) as well. Drinking of alcohol and water was monitored daily/weekly by weighing bottles. From these data, water consumption (ml per kg of body weight per day; ml/kg/day) and alcohol consumption (calculated in g of pure alcohol per kg of body weight per day; g/kg/day) was calculated.

**FIGURE 1:**
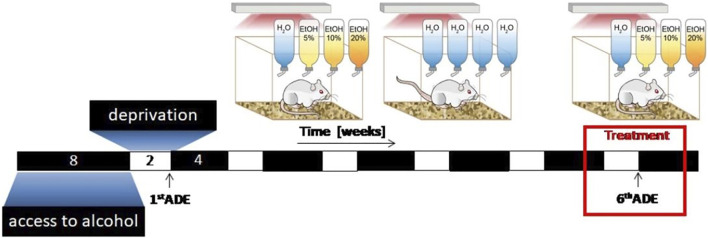
Experimental timeline. All animals underwent repeated cycles of alcohol consumption (in a free choice paradigm with water, 5, 10, or 20% ethanol solutions) and deprivation phases (only water available). After the sixth alcohol deprivation phase pharmacological intervention experiments were conducted. The number eight and four refers to 8 and 4 weeks of alcohol drinking, respectively. The number two stands for a 2 weeks deprivation period.

The long-term voluntary alcohol drinking procedure including all deprivation phases lasted for a duration of 44 weeks. The total time period for concurrent access to alcohol solutions and water was 32 weeks. All animals underwent six 2-week deprivation periods. The first deprivation period was introduced after 8 weeks of continuous alcohol availability. After this deprivation period, rats were given free access to water and to ethanol solutions for 4 weeks. Then, a second 2-week deprivation period was introduced. This 4-week drinking and 2-week deprivation cycle was performed repeatedly over 44 weeks (Figure 1). After an additional 8 weeks of alcohol baseline drinking an experiment on tolerance development was conducted.

### Pharmacological studies

In order to study the effects of drug treatment on the expression of the ADE, rats were divided into groups (*n* = 7–10) in such way that the mean baseline total alcohol intake was approximately the same in each group. Baseline drinking was monitored daily for 1 week. After the last day of baseline measurement, the ethanol bottles were removed from the cages leaving the animals with free access to food and water for 2 weeks. Thereafter, the first nine groups of male rats were subjected to five i. p. Injections (starting at 7p.m. with 12 h intervals) of either vehicle or LY379268 (1 and 3 mg/kg, *n* = 8 in each treatment condition), LY354740 (3 and 10 mg/kg, *n* = 7–10 in each treatment condition). The alcohol bottles were reintroduced after the second injection (at 7a.m. on the 15th day of alcohol deprivation) and the occurrence of an ADE was determined. Three further injections were then given in 12 intervals.

Tolerance development to repeated mGlu2/3 agonist administration was measured in two additional groups of rats (*n* = 7–8 in each treatment condition) treated with either vehicle or 5 mg/kg of LY354740 (this dose was chosen since 3 and 10 mg/kg dose had nearly identical effects on the ADE and the probability to induce unwanted side effects is higher with repeated use of a high dose) during three subsequent ADEs (seventh, eighth and ninth ADE).

Another seven groups of male rats were used to measure effect of PAMs selective for mGlu2 on relapse-like drinking. Three groups were treated with either vehicle or mGlu2 PAM LY487379 (10 and 30 mg/kg, *n* = 7–8 in each treatment condition). For the experiment in female rats, only vehicle or the highest dose of mGlu2 PAM LY487379 (30 mg/kg, *n* = 8–10 in each treatment condition) was applied since a lower dose was not effective in male rats. Combination treatment of mGlu2/3 and PAM was done by simultaneous administration of lower dose of LY487379 (10 mg/kg) and three different doses of LY379268 (0.3, 1 and 3 mg/kg, *n* = 8 in each treatment condition).

The effective dosing of mGlu2/3 ligands was chosen according to a large body of literature in rats and our prior experience with mGlu2/3 agonists (Cannella et al., 2013; Meinhardt et al., 2013; Meinhardt et al., 2021). The alcohol bottles were reintroduced after the second drug administration and the occurrence of an ADE was determined. Total ethanol (g/kg of body weight/day) and water intake (ml/kg of body weight/day) were measured daily for a subsequent week.

### Home cage locomotor activity measurements by the E-motion system

As described in Vengeliene et al. (2018) home cage locomotor activity was monitored by use of an infrared sensor connected to a recording and data storing system (Mouse-E-Motion by Infra-e-motion, Henstedt-Ulzburg, Germany). This system allows-with high accuracy-to detect any sedative effects resulting from the drug treatment. For this purpose, a Mouse-E-Motion device was placed above each cage (30 cm from the bottom) so that the rat could be detected at any position inside the cage. The device was sampling every second whether the rat was moving or not. The sensor could detect body movement of the rat of at least 1.5 cm from one sample point to the successive one. The data measured by each Mouse-E-Motion device were downloaded and processed with Microsoft Excel. Monitoring of locomotor activity started 4 days before drug treatment procedure and was continued for four more post-treatment days. The percentage of each rat’s locomotor activity during and after treatment days was calculated by using the “before treatment” activity data as a reference (Vengeliene et al., 2018).

### Statistics

Data derived from home-cage drinking (total alcohol intake and water intake) and home-cage locomotor activity was analyzed using a two-way ANOVA with repeated measures (factors were: treatment and day/week). Data analysis regarding the effects of treatment on the change in the animals’ body weight was performed using a one-way ANOVA (factor-treatment). Whenever significant differences were found, post-hoc Student Newman Keuls tests were performed. The chosen level of significance was *p* < 0.05.

## Results

### mGlu2/3 agonists reduced relapse-like drinking without any side effects

**FIGURE 2:**
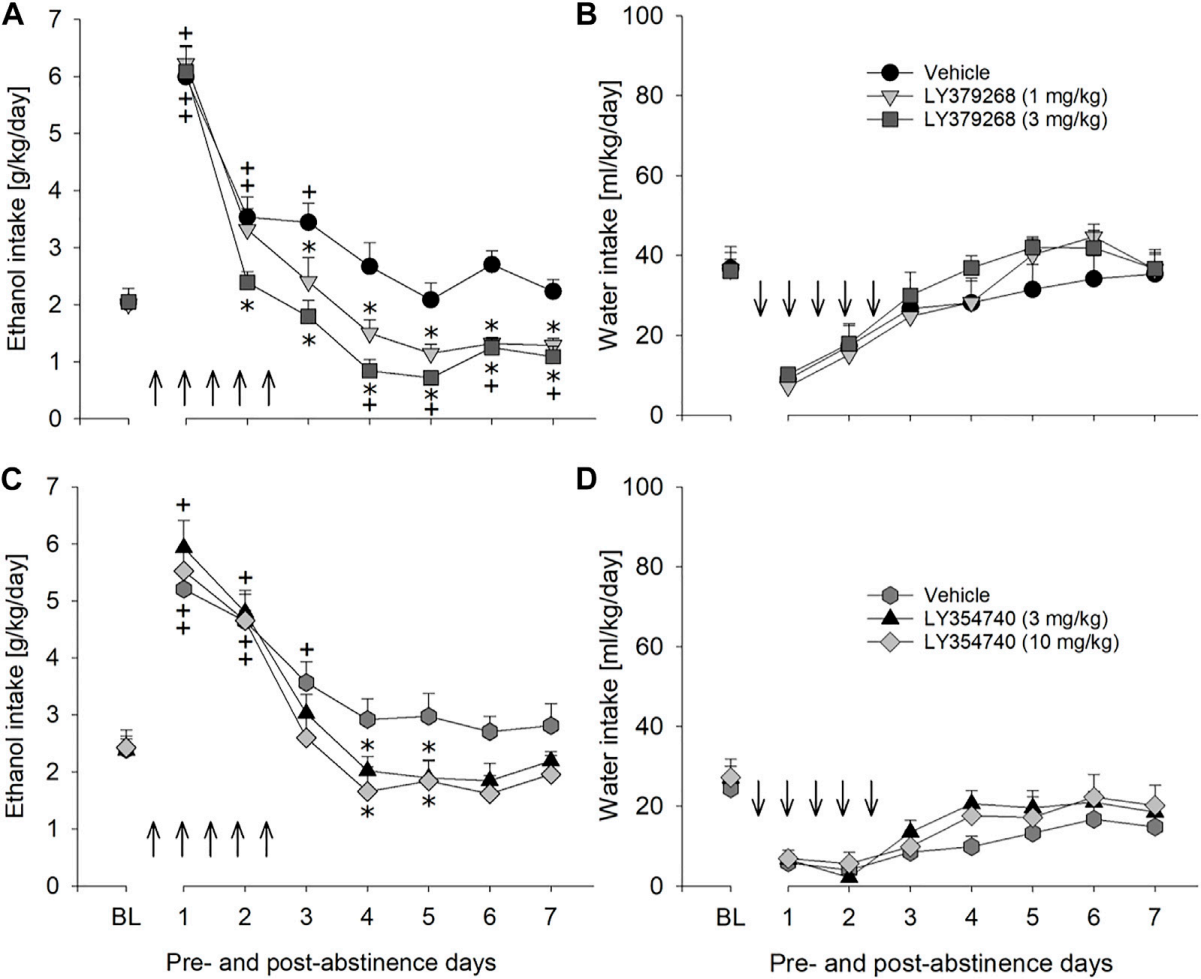
Effects of mGlu2/3 agonists (LY379268 and LY354740) on relapse-like alcohol consumption in male rats. Total alcohol intake (g/kg/day) before (shown as the average of the last 3 days of baseline drinking (BL) and after an alcohol deprivation period of 2 weeks. Arrows indicate a total of five, once every 12 h, administrations of either **(A)** vehicle (*n* = 8), 1 mg/kg of LY379268 (*n* = 8) or 3 mg/kg of LY379268 (*n* = 8), and **(C)** vehicle (*n* = 10), 3 mg/kg of LY354740 (*n* = 8) or 10 mg/kg of LY354740 (*n* = 7). All animals were re-exposed to alcohol immediately after the second drug administration. In **(B)** and **(D)** total water intake (ml/kg/day) before (shown as the average of the last 3 days, BL) and after an alcohol deprivation period of 2 weeks is shown for LY379268 and LY354740, respectively. Data are presented as means ± S.E.M. * indicates significant differences from the vehicle group and + indicates significant difference from baseline, *p* < 0.05.

We used an established rat model of relapse—the alcohol deprivation effect (ADE) model—to test if the mGlu2/3 agonist LY379268 would affect relapse-like drinking. Following the re-introduction of alcohol solutions after a period of abstinence, the vehicle-treated group showed a typical increase in alcohol consumption, indicating the occurrence of an ADE (Figure 2). With respect to the LY379268 treatment, a two-way ANOVA for repeated measures revealed a significant increase in alcohol intake after a deprivation phase in all animal groups as compared to basal drinking [factor day: F_[7,147]_ = 125.2, *p* < 0.001]. The LY379268 treatment caused delayed but significant reduction in the expression of the ADE [factor day × treatment group: F_[14,147]_ = 2.8, *p* < 0.001] (Figure 2A). No significant difference in water intake was seen [factor day × treatment group: *p* = 0.57] (Figure 2B). LY379268 treatment did not lead to significant changes in body weight [factor treatment group: *p* = 0.27] (data not shown), showing that food intake or metabolism was not altered during the treatment days. Locomotor activity data were analyzed using recordings of 12-h post-injection intervals that corresponded animals’ active phase. Overall, there was a general reduction in home-cage activity seen in all animal groups following re-gained access to alcohol [factor day: F_[6,126]_ = 125.2, *p* < 0.001]. However, two-way ANOVA did not reveal significant changes in activity of LY379268 treated animals, when compared to the vehicle-treated rats [factor treatment group: *p* = 0.61 and factor day × treatment group: *p* = 0.15] (Figure 5A). These data together with the recordings of the animal’s body weight suggests that repeated administration of LY379268 in alcohol addicted rats does not lead to any obvious side effects.

Next, we tested LY354740, a structural analog of LY379268 with a six times higher ability to discriminate between mGlu2 and mGluR3. The LY354740 treatment also significantly reduced expression of the ADE [factor day × treatment group: F_[14,154]_ = 2.4, *p* < 0.01] but, similarly to LY379268, this reduction was delayed (Figure 2C). No significant difference in water intake was seen [factor day × treatment group: *p* = 0.60] (Figure 2D). LY354740 treatment did not lead to significant changes in body weight [factor treatment group: *p* = 0.63] (data not shown), showing that food intake or metabolism was not altered during the treatment days. Overall, there was a general reduction in home-cage activity in all animal groups following re-gained access to alcohol [factor day: F_[6,126]_ = 7.8, *p* < 0.001]. However, two-way ANOVA did not show any significant changes in activity of LY354740 treated animals, when compared to the vehicle-treated rats [factor treatment group: *p* = 0.47 and factor day × treatment group: *p* = 0.78] (Figure 5B). Similar to the results with LY379268 treatment, these data show that repeated administration of LY354740 does not lead to nonspecific treatment effects.

### No tolerance development with repeated administration of an mGlu2/3 agonist

**FIGURE 3:**
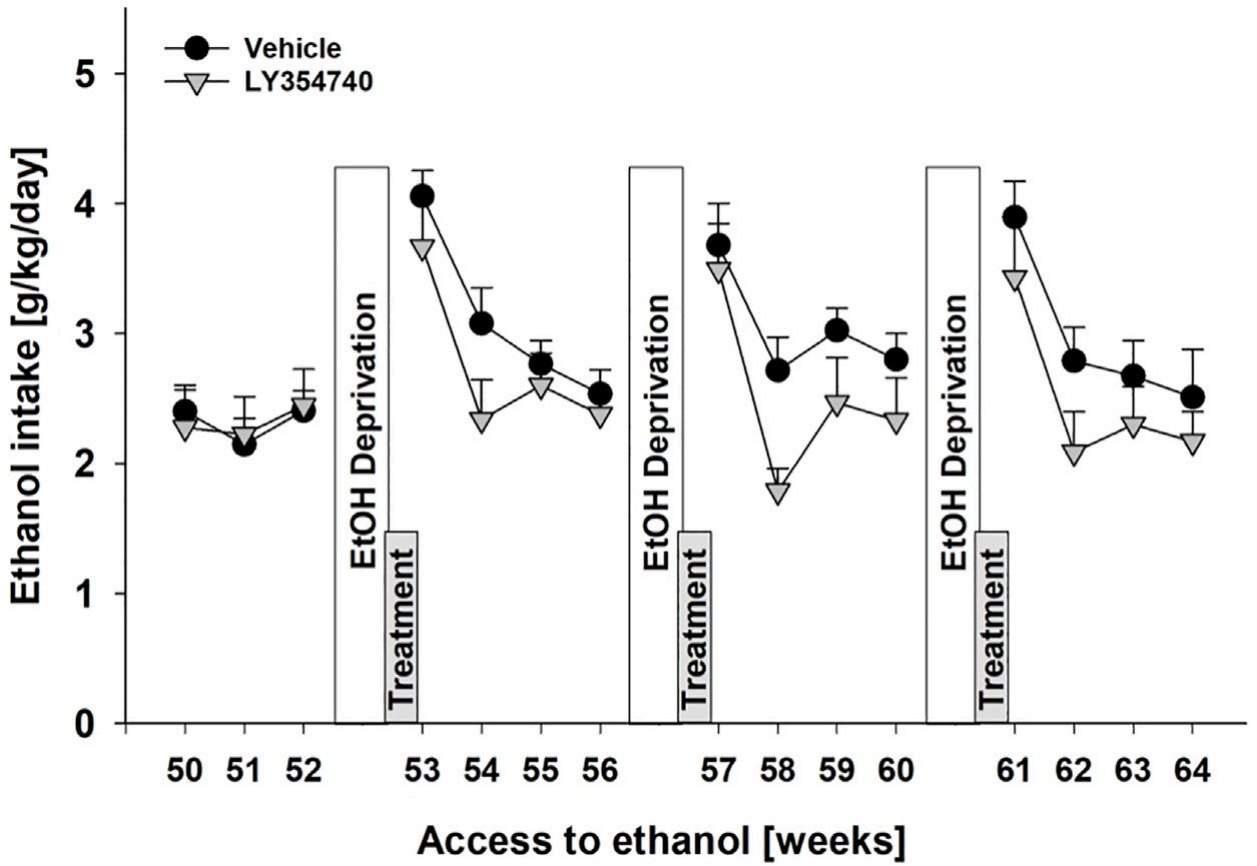
No development of tolerance with repeated administration of mGlu2/3 agonist during relapse-like alcohol consumption in male rats. Alcohol consumption was calculated in g of pure alcohol per kg of body weight per day and presented as the average daily intake during weeks 50–64 of voluntary alcohol drinking interrupted with deprivation phases (seventh, eighth, and ninth EtOH deprivation). Administration of either vehicle (*n* = 8) or 5 mg/kg of LY354740 (*n* = 7) started on the last day of the seventh alcohol deprivation period, which was introduced following week 52 of access to alcohol. Each animal was given five injections every 12 h before and during three subsequent post-abstinence drinking phases. Data are presented as means ± S.E.M.

As is a previous study by us (Vengeliene et al., 2010) subsequent ADE measurements were performed to test for the development of tolerance and to test for persistent treatment effects in a drug-free period. Measurement of weekly alcohol intake showed that alcohol consumption was significantly different over the entire time course of the experiment [factor week: F_[14,182]_ = 16.0, *p* < 0.001] (Figure 3). As a result of repeated LY354740 treatment, weekly alcohol consumption during all post-abstinence weeks and subsequent baseline drinking tended to be lower compared with the vehicle-treated group [factor treatment group: *p* = 0.22 and treatment group × week interaction effect: *p* = 0.33] (Figure 3). These data show that repeated sub-chronic treatment with an mGlu2/3 agonist does not induce tolerance with respect to its anti-relapse properties.

### The mGlu2 PAM LY487379 reduced relapse-like drinking

**FIGURE 4:**
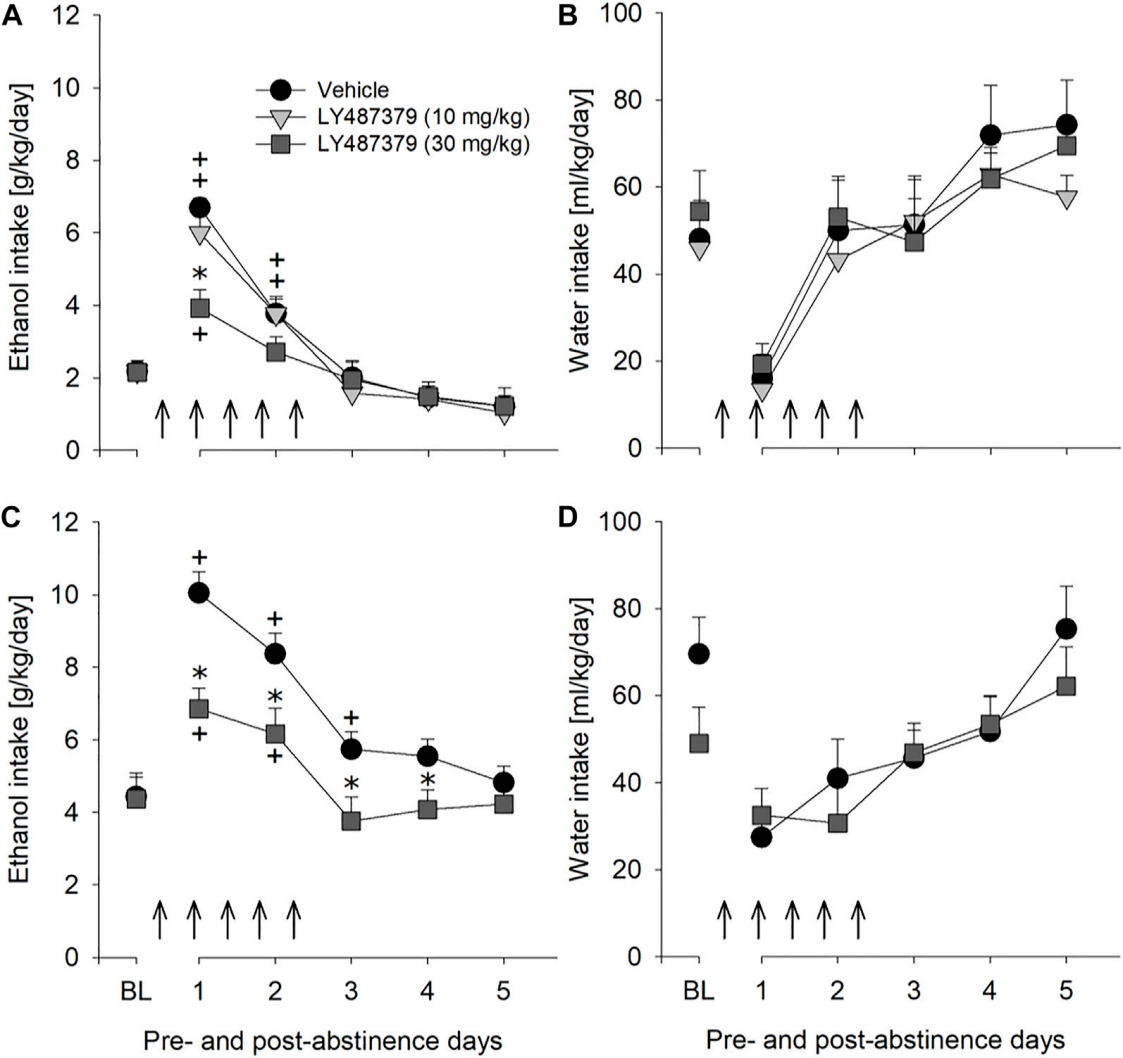
Effects of the mGlu2 PAM LY487379 on relapse-like alcohol consumption in male and female rats and on water consumption in male rats. Total alcohol intake (g/kg/day) before (shown as the average of the last 3 days of baseline drinking (BL) and after an alcohol deprivation period of 2 weeks. Arrows indicate a total of five, once every 12 h, administrations of either **(A)** vehicle (*n* = 8), 10 mg/kg of LY487379 (*n* = 7), or 30 mg/kg of LY487379 (*n* = 7) in male rats, and **(C)** vehicle (*n* = 8) or 30 mg/kg of LY487379 (*n* = 10) in female rats. All animals were re-exposed to alcohol immediately after the second drug administration. In **(B,D)** total water intake (ml/kg/day) before (shown as the average of the last 3 days, BL) and after an alcohol deprivation period of 2 weeks is shown. Data are presented as means ± S.E.M. * indicates significant differences from the vehicle group and + indicates significant difference from baseline, *p* < 0.05.

**FIGURE 5:**
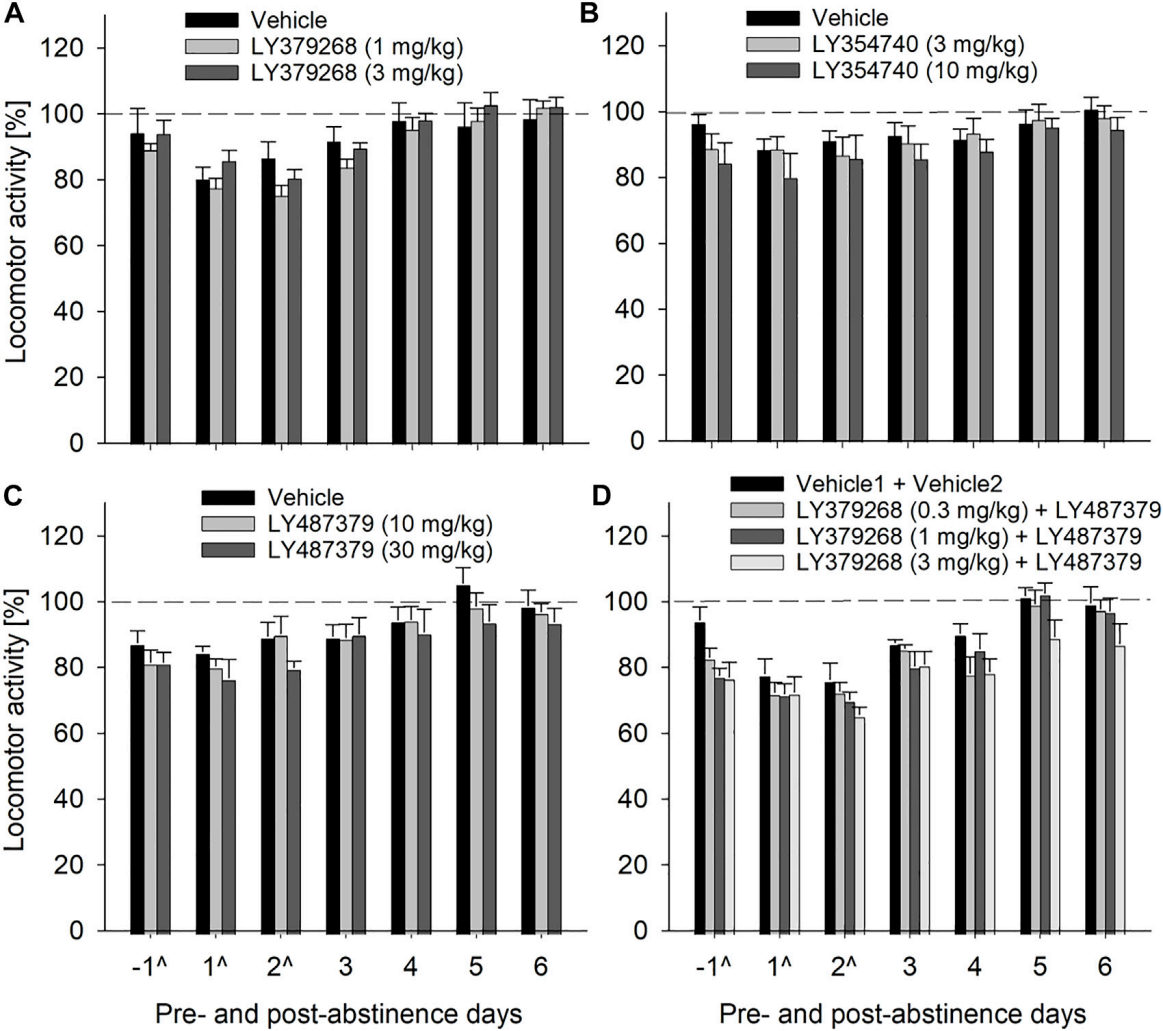
Effects of mGlu2/3 agonists (LY379268 and LY354740), mGlu2 PAM (LY487379) and agonist + PAM combinations on locomotor activity in male rats. Locomotor activity is shown as 12-h post-injection intervals of the animals’ active phase. The percentage of each rat’s locomotor activity during and after treatment days was calculated with respect to basal activity prior to treatment (average of the last 3 days, dashed line). Each animal was given a total of five injections every 12 h (injection days are marked as “^“) of either **(A)** vehicle (*n* = 8), 1 mg/kg of LY379268 (*n* = 8) or 3 mg/kg of LY379268 (*n* = 8) **(B)** vehicle (*n* = 10), 3 mg/kg of LY354740 (*n* = 8) or 10 mg/kg of LY354740 (*n* = 7) **(C)** vehicle (*n* = 8), 10 mg/kg of LY487379 (*n* = 7) or 30 mg/kg of LY487379 (*n* = 7) and **(D)** combination of Vehicle1 (*n* = 8) used for LY379268 with Vehicle2 (*n* = 8) used for LY487379; 0.3 mg/kg of LY379268 with 10 mg/kg of LY487379 (*n* = 8); 1 mg/kg of LY379268 with 10 mg/kg of LY487379 (*n* = 8); and 3 mg/kg of LY379268 with 10 mg/kg LY487379 (*n* = 8). All animals were re-exposed to alcohol immediately after the second drug administration. Data are presented as means ± S.E.M.

Although the results with mGlu2/3 agonists on relapse drinking seemed promising, we used LY487379 as a highly specific PAM that potentiates the effect of glutamate specifically on the mGlu2 subtype. A two-way repeated measures ANOVA showed a significant increase in alcohol intake after a deprivation phase in all groups of male rats as compared to basal drinking [factor day: F_[7,133]_ = 79.1, *p* < 0.001] (Figure 4A). Administration of LY487379 significantly reduced expression of the ADE [factor day × treatment group: F_[14,133]_ = 3.7, *p* < 0.001] (Figure 4A). No significant difference in water intake was seen [factor day × treatment group: *p* = 0.82] (Figure 4B). Treatment of animals with LY487379 did not cause significant loss in body weight [factor treatment group: *p* = 0.09] (data not shown). Overall, there was a general reduction in home-cage activity seen in all animal groups following re-gained access to alcohol [factor day: F_[6,108]_ = 16.1, *p* < 0.001]. However, two-way ANOVA did not show significant changes in activity of LY487379 treated male animals when compared to the vehicle-treated rats [factor treatment group: *p* = 0.39 and factor day × treatment group: *p* = 0.69] (Figure 5C). These data, together with the recordings of the animals’ body weight, suggest that repeated administration of LY487379 in male rats does not lead to occurrence of nonspecific effects.

mGlu2 PAM LY487379 was also evaluated to test the potential for sexually dimorphic effects, since sex differences are often seen in the efficacy of alcoholism treatment (Sanchis-Segura and Becker, 2016). Similarly to male rats, treatment with 30 mg/kg of LY487379 caused significant but longer-lasting reduction in relapse-like drinking during ADE in female rats [factor day × treatment group: F_[5,80]_ = 4.9, *p* < 0.001] (Figure 4C) and no significant changes in water intake [factor day × treatment group: *p* = 0.27] (Figure 4D). This treatment, however, lead to a small but significant decrease in the body weight (by -1.9% compared to the body weight prior to the treatment) [factor treatment group: t_[16]_ = 2.9, *p* < 0.05].

### Treatment with PAM did not potentiate effect of mGlu2/3 agonist on relapse-like drinking but induced some side effects

**FIGURE 6:**
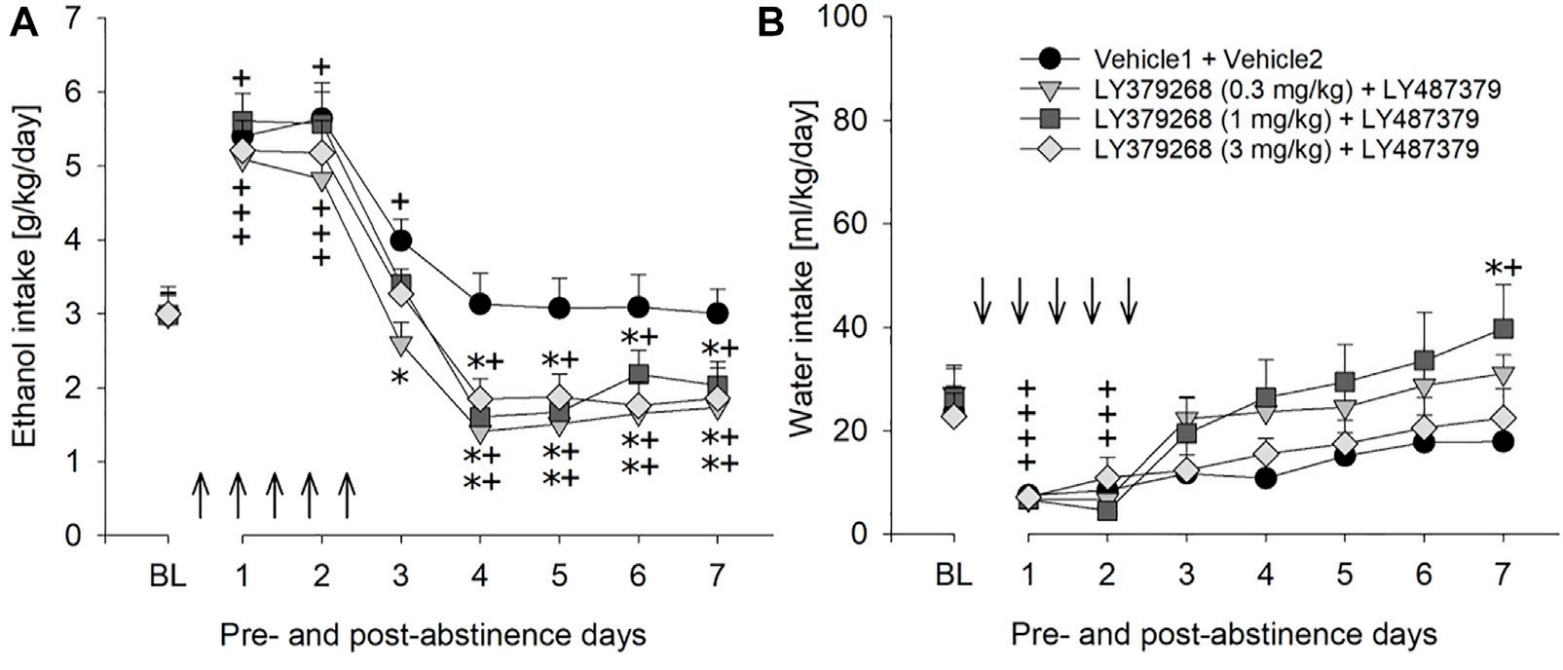
Effects of combined mGlu2/3 agonist (LY379268) and LY487379 treatment on relapse-like alcohol consumption in male rats. Total alcohol intake (g/kg/day) before (shown as the average of the last 3 days of baseline drinking (BL) and after an alcohol deprivation period of 2 weeks. Arrows indicate a total of five, once every 12 h, administrations of either **(A)** combination of Vehicle1 (*n* = 8) used for LY379268 with Vehicle2 (*n* = 8) used for LY487379; 0.3 mg/kg of LY379268 with 10 mg/kg of LY487379 (*n* = 8); 1 mg/kg of LY379268 with 10 mg/kg of LY487379 (*n* = 8); and 3 mg/kg of LY379268 with 10 mg/kg LY487379 (*n* = 8). All animals were re-exposed to alcohol immediately after the second drug administration. In **(B)** total water intake (ml/kg/day) before (shown as the average of the last 3 days, BL) and after an alcohol deprivation period of 2 weeks is shown. Data are presented as means ± S.E.M. * indicates significant differences from the vehicle group and + indicates significant difference from baseline, *p* < 0.05.

Combination treatment of 10 mg/kg of PAM LY487379 (an ineffective dose in the previous experiment) and mGlu2/3 agonist LY379268 was done to see if effects of mGlu2/3 agonists treatment could be potentiated. However, combination treatment affected relapse-like drinking similarly to that seen in animal groups treated with LY379268 alone. Hence, a two-way repeated measures ANOVA showed that combination treatment significantly reduced expression of the ADE [factor day × treatment group: F_[21,196]=_2.1, *p* < 0.01] but similar to mGlu2/3 agonists alone, this reduction was delayed (Figure 6A). Treatment caused significant increase in water intake [factor day × treatment group: F_[21,196]=_1.8, *p* < 0.05], demonstrating a strong selectivity of this combination treatment toward alcohol consumption (Figure 6B), and small (up to –1.5%) but significant loss in body weight [factor treatment group: F_[3,28]=_8.1, *p* < 0.001]. Overall, there was a general reduction in home-cage activity seen in all animal groups following re-gained access to alcohol [factor day: F_[6,156]_ = 36.3, *p* < 0.001]. However, two-way ANOVA also showed significant changes in locomotor activity of LY487379 and LY379268 treated male animals compared to the vehicle-treated rats [factor treatment group: F_[3,156]_ = 4.2, *p* < 0.05 and factor day × treatment group: *p* = 0.44] (Figure 5D). These data, together with the recordings of the animals’ body weight, suggest that repeated administration of LY379268 in male rats may lead to occurrence of non-specific effects.

## Discussion

The present study shows that repeated administration of mGlu2/3 agonists (LY379268 and LY354740) and mGlu2 PAM (LY487379), respectively, reduced alcohol intake under a relapse-like four-bottle free-choice drinking condition in male Wistar rats. Effect of administration of mGlu2/3 agonists on relapse-like alcohol consumption was delayed (i.e., it occurred only on the second-fourth post-abstinence day). Furthermore, treatment of rats with lower dose of LY354740 during three subsequent ADEs revealed that tolerance might not develop to chronic administration of lower doses of mGlu2/3 agonists. Administration of mGlu2 PAM caused a typical reduction of ADE (e.g., Vengeliene et al., 2010), whereby the strongest effect of treatment was measured during the first post-abstinence days. Neither of the abovementioned treatments caused significant changes in water intake, body weight and locomotor activity, showing a good safety profile of these compounds at the dosages used in the study. Female rats responded to mGlu2 PAM treatment stronger than male rats with respect to reduced relapse-like alcohol consumption, so that it lasted for four post-abstinence days (as oppose to 1 day in male rats), however, this treatment also induced significant loss of body weight in these rats. Finally, combination treatment of mGlu2/3 agonist and lower dose of mGlu2 PAM (a dose that was ineffective when given alone) caused significant loss in body weight and reduced locomotor activity of rats but did not augment effect of mGlu2/3 agonist on ADE (or advanced effect of mGlu2/3 agonist towards the first post-abstinence day). However, this combination treatment may have increased the selectivity of mGlu2/3 agonist towards alcohol, considering that reduction of alcohol consumption was accompanied with a significant increase in water intake.

Activation of group II mGlu receptors are known to depress excitatory glutamatergic neurotransmission (Robbe et al., 2002; Grueter and Winder, 2005), which is enhanced specifically under the situation of withdrawal (Hermann et al., 2012) and conditioned withdrawal (Gass et al., 2011). Accordingly, it has been suggested that targeting this group or receptors may help to lower glutamate-induced neurotoxicity and alleviate withdrawal symptoms, and thus reduce risk of alcohol relapse (Holmes et al., 2013). Indeed, the study by Rodd et al. (2006) showed that treatment of alcohol preferring P female rats with mGlu2/3 agonist LY404039 reduced post-abstinence operant alcohol self-administration. Several studies demonstrated that mGlu2/3 receptor agonists suppressed cue-induced reinstatement of alcohol seeking (e.g., Bäckström and Hyytiä, 2005; Meinhardt et al., 2021). In contrast, mGlu2/3 agonists were ineffective in reducing maintenance responding for alcohol, unless used in high doses accompanied by motor-impairing effects (Bäckström and Hyytiä, 2005; Rodd et al., 2006; Besheer et al., 2010). However, in post-dependent rats LY379268 dose-dependently reduced alcohol self-administration (already at a dose of 0.3 mg/kg; Sidhpura et al., 2010). Our study complements these findings demonstrating that two different agonists acting at mGlu2/3 receptors, LY379268 and LY354740, were effective in reducing post-abstinence alcohol consumption in unselected male Wistar rats. However, the data of the present study demonstrated that mGlu2/3 agonist were unable to affect alcohol drinking during the first post-abstinence day. Furthermore, rats maintained significantly lower alcohol consumption during the first post-treatment days, and a trend towards lower alcohol consumption continued during several post-treatment weeks. In our earlier studies, we demonstrated that in long-term drinking rats, repeatedly deprived of alcohol for several weeks, re-exposure to alcohol leads to a loss of control over drinking behavior during the first post-abstinence days. During these days, we measured increased drinking frequency (i.e., increased probability of approaching alcohol bottles), loss of diurnal drinking rhythmicity, and the taste adulteration procedure no longer modified the ADE (Vengeliene et al., 2014). This loss of control was particularly strong during the first day of alcohol re-exposure (Spanagel et al., 1996). Hence, the present study suggests that treatment with mGlu2/3 agonists may not be effective in regaining control over drinking.

It is well known that tolerance to many clinically used compounds (e.g., antidepressants, benzodiazepines, antipsychotics) develops following their repeated administration (e.g., Vinkers and Olivier, 2012). Preclinical addiction research has also demonstrated that compounds effective in reducing drug-related behaviors in animals, such as varenicline, cytisine and acamprosate, lose their efficacy following repeated administration (Cowen et al., 2005; Vengeliene et al., 2010; Sotomayor-Zárate et al., 2013; Spanagel, 2022). Rapid tolerance has also been shown to develop following repeated administration of mGlu2/3 receptor agonists (Jones et al., 2005; Bespalov et al., 2014). In the present study, we showed that low dose of mGlu2/3 receptor agonist LY354740 caused not only long-lasting reduction of alcohol consumption but also this effect was consistently reproduced during each subsequent treatment cycle, suggesting that development of tolerance may not develop to lower doses of group II mGlu receptor agonists.

Finally, mGlu2 PAM LY487379, tested in the ADE model, revealed that behavioral effects of PAM differed from that of mGlu2/3 agonists. Administration of LY487379 caused typical reduction of ADE that was measured already on the very first post-abstinence day (in both male and female rats), showing that this treatment was more effective in restoring control over relapse-like drinking behavior than mGlu2/3 agonists.

Combination treatment using an ineffective dose of PAM (10 mg/kg) and different doses of the mGlu2/3 agonist LY379268 induced some side effects but did not reduce drinking during the first post-abstinence days. This suggests that the PAM cannot increase agonist potency of mGlu2/3 agonists. However, a limitation of this combined treatment experiment is that we did not fully assess whether or not the PAM increases agonist potency. Thus, the combination of the ineffective dose of PAM +0.3 mg/kg dose of LY379268 produced a very similar effect than that of higher agonist doses + PAM. If the 0.3 mg/kg dose of LY379268 constitutes a sub-threshold dose, a synergistic effect would have been obtained. The 0.3 mg/kg dose of LY379268 was not tested alone in the ADE paradigm. However, a previous study by Sidhpura et al. (2010) shows that a 0.3 mg/kg dose of LY379268 can lower ethanol self-administration and stress-induced reinstatement in alcohol dependent rats. We therefore, assume that the 0.3 mg/kg dose of LY379268 produced the effect on the ADE shown in Figure 6A and that the PAM does not increase agonist potency.

The present study demonstrates that mGlu2/3 agonists and LY487379 reduced relapse-like drinking in a well-established and validated rat model with minimal side effects and no development of tolerance. A critical role of mGlu2 in mediating cognitive flexibility has recently been proposed (Meinhardt et al., 2021) and indeed LY487379 treatment can promote cognitive flexibility and facilitate behavioral inhibition (Nikiforuk et al., 2010). Therefore, we suggest that mGlu2 PAM treatment can restore loss of behavioral flexibility in alcohol dependent patients and can thereby reduce the risk for relapse. There is also convincing preclinical evidence that pharmacological mGlu2 stimulation reduces alcohol-seeking behavior (i.e., craving responses) (Kufahl et al., 2011; Augier et al., 2016; Meinhardt et al., 2021). In conclusion, these data provide a rationale for a novel mechanism-based pharmacological intervention that may reduce craving and relapse in alcohol dependent patients.

## Data Availability

The original contributions presented in the study are included in the article/Supplementary Material, further inquiries can be directed to the corresponding author.

## References

[B1] Augier E. Dulman R. S. Rauffenbart C. Augier G. Cross A. J. Heilig M. (2016). The mGluR2 positive allosteric modulator, AZD8529, and cue-induced relapse to alcohol seeking in rats. Neuropsychopharmacology 41 (12), 2932–2940. 10.1038/npp.2016.107 27339394 PMC5061885

[B3] Bäckström P. Hyytiä P. (2005). Suppression of alcohol self-administration and cue-induced reinstatement of alcohol seeking by the mGlu2/3 receptor agonist LY379268 and the mGlu8 receptor agonist (S)-3, 4-DCPG. Eur. J. Pharmacol. 528 (1-3), 110–118. 10.1016/j.ejphar.2005.10.051 16324694

[B4] Besheer J. Grondin J. J. Cannady R. Sharko A. C. Faccidomo S. Hodge C. W. (2010). Metabotropic glutamate receptor 5 activity in the nucleus accumbens is required for the maintenance of ethanol self-administration in a rat genetic model of high alcohol intake. Biol. Psychiatry 67 (9), 812–822. 10.1016/j.biopsych.2009.09.016 19897175 PMC2854174

[B5] Bespalov A. Müller R. Relo A. L. Hudzik T. (2016). Drug tolerance: A known unknown in translational neuroscience. Trends Pharmacol. Sci. 37 (5), 364–378. 10.1016/j.tips.2016.01.008 26935643

[B6] Bossert J. M. Marchant N. J. Calu D. J. Shaham Y. (2013). The reinstatement model of drug relapse: Recent neurobiological findings, emerging research topics, and translational research. Psychopharmacology 229, 453–476. 10.1007/s00213-013-3120-y 23685858 PMC3770775

[B7] Cannella N. Halbout B. Uhrig S. Evrard L. Corsi M. Corti C. (2013). The mGlu2/3 agonist LY379268 induced anti-reinstatement effects in rats exhibiting addiction-like behavior. Neuropsychopharmacology 38, 1–29. 10.1038/npp.2013.106 23624743 PMC3746689

[B8] Cao A. M. Quast R. B. Fatemi F. Rondard P. Pin J. P. Margeat E. (2021). Allosteric modulators enhance agonist efficacy by increasing the residence time of a GPCR in the active state. Nat. Commun. 12 (1), 5426. 10.1038/s41467-021-25620-5 34521824 PMC8440590

[B9] Caprioli D. Justinova Z. Venniro M. Shaham Y. (2018). Effect of novel allosteric modulators of metabotropic glutamate receptors on drug self-administration and relapse: A review of preclinical studies and their clinical implications. Biol. Psychiatry 84, 180–192. 10.1016/j.biopsych.2017.08.018 29102027 PMC5837933

[B10] Cowen M. S. Adams C. Kraehenbuehl T. Vengeliene V. Lawrence A. J. (2005). The acute anti-craving effect of acamprosate in alcohol-preferring rats is associated with modulation of the mesolimbic dopamine system. Addict. Biol. 10, 233–242. 10.1080/13556210500223132 16109584

[B11] Foo J. C. Meinhardt M. W. Skorodumov I. Spanagel R. (2022). Alcohol solution strength preference predicts compulsive-like drinking behavior in rats. Alcohol. Clin. Exp. Res. Online ahead of print. 10.1111/acer.14910 35871774

[B12] Gass J. T. Sinclair C. M. Cleva R. M. Widholm J. J. Olive M. F. (2011). Alcohol-seeking behavior is associated with increased glutamate transmission in basolateral amygdala and nucleus accumbens as measured by glutamate-oxidase-coated biosensors. Addict. Biol. 16, 215–228. 10.1111/j.1369-1600.2010.00262.x 21054692 PMC3058760

[B13] Grueter B. A. Winder D. G. (2005). Group II and III metabotropic glutamate receptors suppress excitatory synaptic transmission in the dorsolateral bed nucleus of the stria terminalis. Neuropsychopharmacology 30, 1302–1311. 10.1038/sj.npp.1300672 15812571

[B14] Heinz A. Kiefer F. Smolka M. N. Endrass T. Beste C. Beck A. (2020). Addiction Research Consortium: Losing and regaining control over drug intake (ReCoDe)-From trajectories to mechanisms and interventions. Addict. Biol. 25 (2), e12866. 10.1111/adb.12866 31859437

[B15] Hermann D. Weber-Fahr W. Sartorius A. Hoerst M. Frischknecht U. Tunc-Skarka N. (2012). Translational magnetic resonance spectroscopy reveals excessive central glutamate levels during alcohol withdrawal in humans and rats. Biol. Psychiatry 71, 1015–1021. 10.1016/j.biopsych.2011.07.034 21907974

[B16] Holmes A. Spanagel R. Krystal J. H. (2013). Glutamatergic targets for new alcohol medications. Psychopharmacology 229, 539–554. 10.1007/s00213-013-3226-2 23995381 PMC3811052

[B17] Jones C. K. Eberle E. L. Peters S. C. Monn J. A. Shannon H. E. (2005). Analgesic effects of the selective group II (mGlu2/3) metabotropic glutamate receptor agonists LY379268 and LY389795 in persistent and inflammatory pain models after acute and repeated dosing. Neuropharmacology 49 (1), 206–218. 10.1016/j.neuropharm.2005.05.008 15998527

[B18] Kufahl P. R. Martin-Fardon R. Weiss F. (2011). Enhanced sensitivity to attenuation of conditioned reinstatement by the mGluR 2/3 agonist LY379268 and increased functional activity of mGluR 2/3 in rats with a history of ethanol dependence. Neuropsychopharmacology 36 (13), 2762–2773. 10.1038/npp.2011.174 21881571 PMC3230501

[B19] Marino M. J. Conn P. J. (2006). Glutamate-based therapeutic approaches: Allosteric modulators of metabotropic glutamate receptors. Curr. Opin. Pharmacol. 6, 98–102. 10.1016/j.coph.2005.09.006 16368268

[B20] Meinhardt M. W. Hansson A. C. Perreau-Lenz S. Bauder-Wenz C. Stählin O. Heilig M. (2013). Rescue of infralimbic mGluR2 deficit restores control over drug-seeking behavior in alcohol dependence. J. Neurosci. 33, 2794–2806. 10.1523/JNEUROSCI.4062-12.2013 23407939 PMC3711176

[B21] Meinhardt M. W. Pfarr S. Fouquet G. Rohleder C. Meinhardt M. L. Barroso-Flores J. (2021). Psilocybin targets a common molecular mechanism for cognitive impairment and increased craving in alcoholism. Sci. Adv. 7 (47), eabh2399. 10.1126/sciadv.abh2399 34788104 PMC8598005

[B22] Meinhardt M. W. Sommer W. H. (2015). Postdependent state in rats as a model for medication development in alcoholism. Addict. Biol. 20, 1–21. 10.1111/adb.12187 25403107

[B23] Nikiforuk A. Popik P. Drescher K. van Gaalen M. Relo A. L. A-L. Mezler M. (2010). Effects of a positive allosteric modulator of group II metabotropic glutamate receptors, LY487379, on cognitive flexibility and impulsive-like responding in rats. J. Pharmacol. Exp. Ther. 335, 665–673. 10.1124/jpet.110.170506 20739457

[B25] Robbe D. Alonso G. Chaumont S. Bockaert J. Manzoni O. J. (2002). Role of p/q-Ca2+ channels in metabotropic glutamate receptor 2/3-dependent presynaptic long-term depression at nucleus accumbens synapses. J. Neurosci. 22 (11), 4346–4356. 10.1523/JNEUROSCI.22-11-04346.2002 12040040 PMC6758789

[B26] Rodd Z. A. McKinzie D. L. Bell R. L. McQueen V. K. Murphy J. M. Schoepp D. D. (2006). The metabotropic glutamate 2/3 receptor agonist LY404039 reduces alcohol-seeking but not alcohol self-administration in alcohol-preferring (P) rats. Behav. Brain Res. 171 (2), 207–215. 10.1016/j.bbr.2006.03.032 16678921

[B27] Sanchis-Segura C. Becker J. B. (2016). Why we should consider sex (and study sex differences) in addiction research. Addict. Biol. 21 (5), 995–1006. 10.1111/adb.12382 27029841 PMC5585537

[B28] Schaffhauser H. Rowe B. A. Morales S. Chavez-Noriega L. E. Yin R. Jachec C. (2003). Pharmacological characterization and identification of amino acids involved in the positive modulation of metabotropic glutamate receptor subtype 2. Mol. Pharmacol. 64, 798–810. 10.1124/mol.64.4.798 14500736

[B29] Schoepp D. D. Johnson B. G. Wright R. A. Salhoff C. R. Mayne N. G. Wu S. (1997). LY354740 is a potent and highly selective group II metabotropic glutamate receptor agonist in cells expressing human glutamate receptors. Neuropharmacology 36 (1), 1–11. 10.1016/s0028-3908(96)00160-8 9144636

[B30] Sidhpura N. Weiss F. Martin-Fardon R. (2010). Effects of the mGlu2/3 agonist LY379268 and the mGlu5 antagonist MTEP on ethanol seeking and reinforcement are differentially altered in rats with a history of ethanol dependence. Biol. Psychiatry 67 (9), 804–811. 10.1016/j.biopsych.2010.01.005 20189165 PMC2854322

[B31] Sotomayor-Zárate R. Gysling K. Busto U. E. Cassels B. K. Tampier L. Quintanilla M. E. (2013). Varenicline and cytisine: Two nicotinic acetylcholine receptor ligands reduce ethanol intake in university of Chile bibulous rats. Psychopharmacology 227, 287–298. 10.1007/s00213-013-2974-3 23344555

[B32] Spanagel R. (2017). Animal models of addiction. Dialogues Clin. Neurosci. 19, 247–258. 10.31887/dcns.2017.19.3/rspanagel 29302222 PMC5741108

[B33] Spanagel R. (2022). Ten points to improve reproducibility and translation of animal research. Front. Behav. Neurosci. 16, 869511. 10.3389/fnbeh.2022.869511 35530730 PMC9070052

[B34] Spanagel R. Hölter S. Allingham K. Landgraf R. Zieglgänsberger W. (1996). Acamprosate and alcohol: I. Effects on alcohol intake following alcohol deprivation in the rat. Eur. J. Pharmacol. 305, 39–44. 10.1016/0014-2999(96)00174-4 8813529

[B35] Spanagel R. Durstewitz D. Hansson A. Heinz A. Kiefer F. Köhr G. Matthäus F. Nöthen M. M. Noori H. R. Obermayer K. Rietschel M. Schloss P. Scholz H. Schumann G. Smolka M. Sommer W. Vengeliene V. Walter H. Wurst W. Zimmermann U. S. Stringer S. Smits Y. Derks E. M. Addiction GWAS Resource Group (2013). A systems medicine research approach for studying alcohol addiction. Addict. Biol. 18 (6), 883–896. 10.1111/adb.12109 24283978

[B36] Spanagel R. Hölter S. M. (1999). Long-term alcohol self-administration with repeated alcohol deprivation phases: An animal model of alcoholism? Alcohol Alcohol 34, 231–243. 10.1093/alcalc/34.2.231 10344783

[B37] Spanagel R. Vengeliene V. (2013). New pharmacological treatment strategies for relapse prevention. Curr. Top. Behav. Neurosci. 13, 583–609. 10.1007/7854_2012_205 22389180

[B38] Vengeliene V. Celerier E. Chaskiel L. Penzo F. Spanagel R. (2009). Compulsive alcohol drinking in rodents. Addict. Biol. 14 (4), 384–396. 10.1111/j.1369-1600.2009.00177.x 19740366

[B39] Vengeliene V. Leonardi-Essmann F. Sommer W. H. Marston H. M. Spanagel R. (2010). Glycine transporter-1 blockade leads to persistently reduced relapse-like alcohol drinking in rats. Biol. Psychiatry 68, 704–711. 10.1016/j.biopsych.2010.05.029 20655511

[B41] Vengeliene V. Bilbao A. Spanagel R. (2014). The alcohol deprivation effect model for studying relapse behavior: A comparison between rats and mice. Alcohol 48 (3), 313–320. 10.1016/j.alcohol.2014.03.002 24811155

[B42] Vengeliene V. Moeller A. Meinhardt M. W. Beardsley P. M. Sommer W. H. Spanagel R. (2016). The calpain inhibitor A-705253 attenuates alcohol-seeking and relapse with low side-effect profile. Neuropsychopharmacology 41 (4), 979–988. 10.1038/npp.2015.225 26216521 PMC4748423

[B43] Vengeliene V. Roßmanith M. Takahashi T. T. Alberati D. Behl B. Bespalov A. (2018). Targeting Glycine reuptake in alcohol seeking and relapse. J. Pharmacol. Exp. Ther. 365 (1), 202–211. 10.1124/jpet.117.244822 29367277

[B44] Vinkers C. H. Olivier B. (2012). Mechanisms underlying tolerance after long-term benzodiazepine use: A future for subtype-selective GABA(A) receptor modulators? Adv. Pharmacol. Sci. 2012, 416864. 10.1155/2012/416864 22536226 PMC3321276

[B45] Zhao Y. Dayas C. V. Aujla H. Baptista M. A. Martin-Fardon R. Weiss F. (2006). Activation of group II metabotropic glutamate receptors attenuates both stress and cue-induced ethanol-seeking and modulates c-fos expression in the hippocampus and amygdala. J. Neurosci. 26 (39), 9967–9974. 10.1523/JNEUROSCI.2384-06.2006 17005860 PMC6674480

[B46] Zhou Z. Karlsson C. Liang T. Xiong W. Kimura M. Tapocik J. D. (2013). Loss of metabotropic glutamate receptor 2 escalates alcohol consumption. Proc. Natl. Acad. Sci. U. S. A. 110 (42), 16963–16968. 10.1073/pnas.1309839110 24082084 PMC3800985

